# Promoting Linguistically Based Health Literacy in Doctors for Better Cancer Healthcare in Cyprus

**DOI:** 10.1007/s13187-024-02406-z

**Published:** 2024-02-15

**Authors:** Georgios P. Georgiou, Polina Mackay

**Affiliations:** 1 Department of Languages and Literature, University of Nicosia, Nicosia, Cyprus; 2 Phonetic Lab, University of Nicosia, Nicosia, Cyprus

**Keywords:** Linguistics, Training, Health literacy, Doctors, Cyprus

## Abstract

This paper explores a novel approach in cancer healthcare, underscoring the significance of fostering health literacy among doctors by enhancing their ability to employ linguistically oriented strategies to extract and interpret the linguistic behavior of patients. The focus is on the Greek-Cypriot context, where research on health literacy is still in its nascent stages.

Cyprus is a small island country situated in the Mediterranean Sea and is a member of the European Union (EU). The country’s General Healthcare System (GHS) is one of the youngest in the EU, beginning in 2019. It now offers health services free of charge to all Cypriot citizens, EU citizens, and eligible third-country nationals (Cyprus health system information and resources, n.d.) [1].

Despite the increasing good healthcare provision, cancer is on the rise, becoming one of the most significant causes of death in the country. There are more than 4000 new cases every year in a population of less than a million [2]. An emerging and pivotal aspect of cancer care pertains to the health literacy (HL) of patients. HL is defined as an individual’s ability to access, comprehend, evaluate, and effectively utilize health-related information. Research shows that lower levels of HL are associated with unfavorable clinical outcomes for cancer patients including reduced engagement in screening and preventive measures, delayed recognition of symptoms and seeking medical assistance, and limited awareness about cancer, its prevention, and treatment, among others [3]. The importance of developing HL in the Greek-Cypriot context has only recently gained recognition, and there is currently a limited number of studies addressing this matter.

Doctors often face a significant challenge in understanding the linguistic behavior of their patients. This can stem from various sources, including patients struggling to articulate their symptoms due to pain, speech difficulties resulting from cancer treatments, and difficulty in expressing themselves coherently or recalling the right words due to emotional distress among other factors. As an illustration, patients might exhibit compromised pronunciation, creating challenges for doctors in comprehending their symptoms, addressing concerns, or understanding requests. These difficulties extend to the appropriate use of syntax and vocabulary during patient-doctor interaction. Overcoming these challenges becomes feasible when doctors employ specific strategies aimed at extracting precise linguistic information from patients. In recent years, there has been a growing number of voices advocating for improved treatment and heightened awareness of these issues. For instance, patients from the breast cancer community in Cyprus report a particular difficulty relating to the lack of linguistically oriented strategies on the part of doctors to help them express themselves more clearly (Life Stories, 2017) [4].

These reports underscore the need to bolster HL among doctors, extending beyond the psychological support they provide. It is vital to emphasize that the contemporary definition of HL is more expansive than it has been in the past. HL is a dynamic and continuously evolving concept that also encompasses healthcare providers [5], highlighting the need to develop such skills within this population. A novel and holistic approach is required, one that focuses on equipping doctors with linguistically oriented HL training. Such training should emphasize the development of specific strategies aimed at enhancing the clinical outcomes of cancer patients. For example, doctors should be trained in understanding the context of a patient’s language to decipher words or phrases that might be pronounced unclearly by the patients or breaking down complex words into simpler syllables to help patients pronounce them more clearly. Furthermore, training should include encouraging patients to rephrase sentences for enhanced clarity, actively listening to patients, particularly when they encounter difficulty articulating thoughts, refraining from interruptions to allow for the expression of emotions, and incorporating well-timed pauses among other effective techniques. Merely offering basic instructions to doctors is insufficient; instead, there is a need to implement systematically structured training sessions that specifically concentrate on linguistic aspects. These sessions could seamlessly integrate into the regular curriculum for doctors, facilitated by professionals such as linguists and communication scientists.

In this paper, we approach the investigation of HL among doctors in an underresearched setting from a novel perspective, which relies on the employment of linguistically oriented strategies for the extraction of linguistic information from patients. Doctors are tasked with the responsibility of upholding elevated HL skills to overcome communication barriers with their patients, ultimately leading to enhanced outcomes in cancer care. In addition, the state bears the responsibility of ensuring the provision of top-quality healthcare for cancer patients, both within Cyprus and beyond its borders. This can take place not only through the explicit implementation of linguistic training for doctors but also through the inclusion and/or refinement of pertinent material within the educational curricula of medical studies.

## Data Availability

There is no data related to this article.
